# Draft Genome Sequences of Two Novel *Acidimicrobiaceae* Members from an Acid Mine Drainage Biofilm Metagenome

**DOI:** 10.1128/genomeA.01563-15

**Published:** 2016-01-14

**Authors:** Ameet J. Pinto, Jonathan O. Sharp, Michael J. Yoder, Robert Almstrand

**Affiliations:** a Infrastructure and Environment Research Division, School of Engineering, University of Glasgow, Glasgow, United Kingdom; b Department of Civil and Environmental Engineering, Colorado School of Mines, Golden, Colorado, USA

## Abstract

Bacteria belonging to the family *Acidimicrobiaceae* are frequently encountered in heavy metal-contaminated acidic environments. However, their phylogenetic and metabolic diversity is poorly resolved. We present draft genome sequences of two novel and phylogenetically distinct *Acidimicrobiaceae* members assembled from an acid mine drainage biofilm metagenome.

## GENOME ANNOUNCEMENT

Members of the family *Acidimicrobiaceae*, with the exception of *Ilumatobacter* sp., are typically found in acidic, metal laden environments where they characteristically oxidize ferrous iron (1). Although of interest for environmental applications (2), there are limited data on the phylogenetic and metabolic diversity within this family. Of the five *Acidimicrobiaceae* genome assemblies available, only two are complete and annotated (*Acidimicrobium ferrooxidans* DSM 10331 [3] and *Ilumatobacter coccineus* YM16-304 [4]). We present two additional, >90% complete draft genomes of novel *Acidimicrobiaceae* members, designated RAAP-2 and RAAP-3, from an acid-mine drainage (AMD) metagenome originating from a streamer biofilm growing in acidic (pH ~ 3) heavy metal-contaminated mine-water in Colorado, USA. DNA was extracted using the Power Soil DNA isolation kit (Mo Bio Laboratories, Carlsbad, CA, USA). The genomic DNA library was prepared using an Illumina TruSEQ DNA library kit and sequenced on an Illumina HiSEQ 2500 paired end flow cell (2 × 125-bp read length, V4 Chemistry) at the Genomics and Microarray Core, University of Colorado, Denver. Reads were co-assembled with four additional samples using IDBA-UD (5), followed by binning of scaffolds using CONCOCT (6). Reads mapping to scaffolds within each genome bin were extracted and reassembled using IDBA-UD. Post-reassembly, contigs less than 1 kb, and coverage profile outliers were removed. This resulted in a draft genome size of 2.24 MB for RAAP-2 and 3.05 MB for RAAP-3 with 58 and 149 contigs and G+C content of 65 and 47%, respectively. CheckM (7) estimated the completeness of the genomes to be 91.5% and 98.3%, respectively, with <1.5% contamination. The two draft genomes shared 70.6% average nucleotide identity with each other (genome-to-genome distance calculator [http://ggdc.dsmz.de/]). The genomes were annotated using Prodigal (8) and RAPSearch2 (9) to identify best matches in the KEGG database (10). RAAP-2 and RAAP-3 consisted of 2,174 and 2,668 coding DNA sequences with 2,022 and 2,244 matches to the KEGG database, respectively. AMPHORA2 (11) indicated the phylogenetic placement of these two genome bins as *Acidimicrobium*. A phylogenetic tree was constructed using a concatenated alignment of 16 ribosomal proteins (12) using 66 representative genomes from the phylum *Actinobacteria*. This indicated that RAAP-2 should be placed between *A. ferrooxidans* and *I. coccineus*, whereas RAAP-3 was more closely related to *A. ferrooxidans*. Noticeable metabolic differences between these draft genomes and *A. ferrooxidans* include a ferric iron transport system absent in *A. ferrooxidans*. Moreover, RAAP-2 contains an additional iron complex transport system and a complete pathway for assimilatory sulfate reduction. This pathway is absent in RAAP-3, which is more closely related to isolate DSM 10331 derived from geothermal hot springs (13) where steam vents constantly supply H_2_S (14), hence reducing the benefit of assimilatory sulfate reduction. These draft genomes provide additional information on the ecology, diversity, and metabolic potential of *Acidimicrobiaceae*, which is beneficial for expanding our knowledge of microbial ecology in metal-contaminated acidic waters.

### Nucleotide sequence accession numbers.

This whole-genome shotgun project has been deposited at GenBank under accession numbers LMAD00000000 (RAAP-2) and LMAE00000000 (RAAP-3). The versions described in this paper are LMAD01000000 and LMAE01000000, respectively.
